# Sustainable Flow‐Synthesis of (Bulky) Nucleoside Drugs by a Novel and Highly Stable Nucleoside Phosphorylase Immobilized on Reusable Supports

**DOI:** 10.1002/cssc.202102030

**Published:** 2021-11-27

**Authors:** Ana I. Benítez‐Mateos, Francesca Paradisi

**Affiliations:** ^1^ Department of Chemistry Biochemistry and Pharmaceutical Sciences University of Bern Freiestrasse 3 3012 Bern Switzerland

**Keywords:** enzymes, enzyme immobilization, flow biocatalysis, nucleosides, purine nucleoside phosphorylase

## Abstract

The continuous synthesis of valuable nucleoside drugs was achieved in up to 99 % conversion by using a novel halotolerant purine nucleoside phosphorylase from *Halomonas elongata* (HePNP). HePNP showed an unprecedented tolerance to DMSO, usually required for substrate solubility, and could be immobilized on agarose microbeads through disulfide bonds, via a genetically fused Cystag. This covalent yet reversible binding chemistry showcased the reusability of agarose microbeads in a second round of enzyme immobilization with high reproducibility, reducing waste and increasing the sustainability of the process. Finally, the flow synthesis of a Nelarabine analogue (6‐*O*‐methyl guanosine) was optimized to full conversion on a 10 mm scale within 2 min residence time, obtaining the highest space‐time yield (89 g L^−1^ h^−1^) reported to date. The cost‐efficiency of the system was further enhanced by a catch‐and‐release strategy that allowed to recover and recirculate the excess of sugar donor from the downstream water waste.

## Graphical abstract

**Nucleoside synthesis**: A novel purine nucleoside phosphorylase (HePNP) from *Halomonas elongata* is characterized, showing a high stability towards co‐solvents. HePNP is immobilized by reversible covalent bonds, allowing the reuse of the support for another immobilization cycle once HePNP becomes inactive. The immobilized HePNP is integrated into a flow reactor for the synthesis of (bulky) nucleoside drugs, also enabling the separation and recycling of the sugar donor excess.

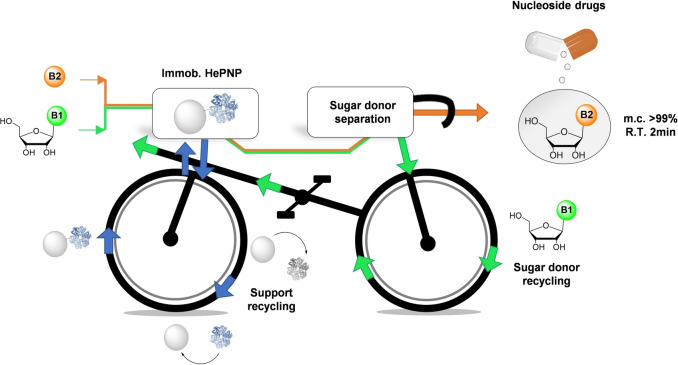

## Introduction

Nucleoside analogues comprise an important family of drugs to treat cancer and viral diseases.^[1]^ Indeed, some of the nucleoside analogues are categorized as orphan drugs (i. e., Nelarabine); medicines used to treat rare diseases. Pharmaceutical companies have limited interest in the production of orphan drugs due to the spiraling costs of drug development and production and the low return on investment, which are a major handicap for their industrial production.^[2]^ Not all nucleoside analogues are of course classified as orphan drugs, but even in the case of high‐demand molecules, more sustainable and efficient synthetic methods are highly sought after to reduce costs and environmental impact.^[3]^ Traditionally, the synthesis of nucleoside analogues has been addressed with conventional synthetic chemistry. Nonetheless, the low selectivity of this approach together with the multiple complex steps and the use of protecting groups, which may generate hazardous chemical by‐products, have prompted the search for alternative synthetic methods.^[4]^

**Scheme 1:**
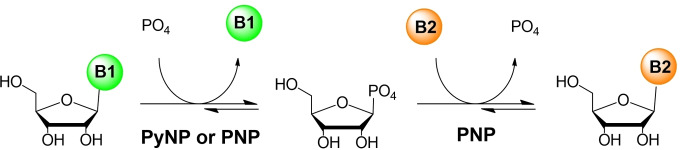
One‐pot enzymatic phosphorolysis‐transglycosylation reaction for the synthesis of nucleoside analogues. The first reaction step may be catalyzed by a pyrimidine nucleoside phosphorylase (PyNP) or a purine nucleoside phosphorylase (PNP) depending on the nucleobase (B1) of the sugar donor. B2 corresponds to a purine nucleobase.

Enzymatic transformations are an appealing strategy due to their great enantio‐, stereo‐, and regioselectivity. Enzymes are also biocompatible and biodegradable, making the process more sustainable and cost‐efficient.^[5]^ Nucleoside phosphorylases (EC 2.4 and EC 2.7.7) are a class of enzymes that have been intensively studied during the last 20 years for application in the synthesis of nucleoside analogues.^[6]^ Specifically, purine nucleoside phosphorylases (PNP) are interesting biocatalysts because they have a broad substrate spectrum. The preparation of purine nucleoside drugs has been achieved by using either a single PNP^[7]^ (Scheme 1) or by coupling a PNP with other enzymes such as uridine phosphorylases (UP).[^8^, ^9^, ^10^, ^11^] However, the low water solubility of the nucleosides challenges the intensification of the process.

Enzyme immobilization on solid supports usually confers a higher stability (pH, solvent, temperature) of the biocatalyst,[^12^, ^13^] which can then be easily separated from the reaction media for reutilization or integrated in continuous flow reactors.^[14]^ Enzymes can be immobilized through reversible (i. e., ionic interactions, hydrophobic forces) or irreversible bonds (i. e., covalent bonds) to the support material. Whilst reversible binding chemistries generally provide a better activity of the immobilized enzyme and could in theory enable the reuse of the support material, irreversible immobilization protocols are frequently preferred to avoid the lixiviation of the enzyme, especially in continuous processing. Flow biocatalysis has in fact become a powerful tool to intensify biotransformations.[^15^, ^16^, ^17^] Some examples of nucleoside synthesis by PNP integrated in flow reactors have been reported recently.[^9^, ^11^, ^18^] However, the very low productivities, the poor enzyme stability in presence of co‐solvents, and the still high waste generation limit an implementation at industrial scale.

In this work, we have characterized a novel PNP that shows a high tolerance and stability in presence of co‐solvents. We have investigated the substrate scope of this enzyme, with a special focus on pharmaceutically relevant nucleosides and bulky nucleobases. In agreement with Kaspar et al., maximum yields in NP‐catalyzed reactions can be accomplished independently of the enzyme used; therefore efforts must be focused on identification of novel enzymes that show a higher stability and accept bulky substrates, broadening the chemical scope of NP‐catalyzed reactions.^[19]^ Furthermore, the immobilization of the enzyme has been studied using different material supports and immobilization chemistries. A covalent but reversible immobilization technique has been newly developed for this work, merging the advantages of the reversible binding (reuse of support material, good activity of immobilized enzyme) with those of the irreversible ones (strong interaction, no lixiviation of the enzyme downstream). The immobilized biocatalyst has been integrated in a continuous flow reactor to intensify the production of nucleoside‐based pharmaceuticals. Finally, to further improve the sustainability of the process, the recycling of the substrate, as well as the support material for the immobilization has been explored.

## Results and Discussion

### Identification and characterization of a purine nucleoside phosphorylase from *Halomonas elongata*

The biosynthesis of nucleoside analogues at large scale is limited by the poor water‐solubility of most nucleosides and the low stability of biocatalysts in organic solvents. The halotolerant organism *Halomonas elongata* has been previously exploited in our group as a source of new biocatalysts that easily express in *E. coli* and present a high co‐solvent stability.[^20^, ^21^, ^22^] We identified the annotated gene *deoD*, which codes for a purine nucleoside phosphorylase (EC 2.4.2.1), and cloned it into the plasmid pET28b(+) harbouring a (6x)His‐tag in the C‐terminal and a (6x)Cys‐tag in the N‐terminal for purification and immobilization purposes (Figure S1).

The purine nucleoside phosphorylase from *Halomonas elongata* (HePNP) was expressed in *E. coli* as a 32 kDa protein as observed by SDS‐PAGE (Figure S2). The structure of HePNP was analyzed using a homology model based on the PNP from *Y. tuberculosis* (PDB: 3OCC) with which HePNP shares 72.6 % identity (Figure S3).^[23]^ A hexameric structure is predicted for HePNP as other bacterial PNP of the family I (Figure S4).^[6]^

The purified HePNP showed a maximum phosphorolysis activity of 43 U mg^−1^ with inosine at 60 °C in 100 mm phosphate buffer at pH 7.5 (Figure S5A–C). However, the temperature for the activity assay and biotransformations was decreased to 37 °C to preserve the enzyme activity for longer times. In addition, HePNP fully retained its activity in presence of 10 % of co‐solvents such as acetonitrile (MeCN), dimethylformamide (DMF), dimethylsulfoxide (DMSO), isopropanol (IprOH), methanol (MeOH), and ethanol (EtOH). In comparison with a previously characterized PNP from *A. hydrophila*, the tolerance to 10 % of DMF and 10 % of MeCN was similar.^[8]^ Noteworthy, the activity of HePNP was reduced only 50 % when the content of DMSO was upped to 50 %, while it decreased more than 75 % in presence of 50 % of other co‐solvents (Figure S5D).

### Substrate scope and catalytic efficiency of HePNP

Different nucleosides were submitted to phosphorolysis by HePNP. The enzyme was able to catalyze the phosphorolysis of both 6‐amino and 6‐oxopurine (deoxy)ribonucleosides (Table 1). Moreover, HePNP showed traces of activity with pyrimidine nucleosides, which is an interesting feature that some PNP possess.^[6]^

**Table 1:** Phosphorolysis activity of HePNP with purine and pyrimidine nucleosides.^[a]^

Substrate	Specific activity [U mg^−1^]	Phosphorolysis after 4 h [%]
inosine	20±0.4	57±2
2′‐deoxyinosine	44.9±1.5	54±3
adenosine	11±0.6	40±1
2′‐deoxyadenosine	20.4±2.1	45±1
guanosine	23.9±0.2	23±1
thymidine	1.2±0.1	4±0
5‐methyluridine	n.d.^[b]^	n.d.
uridine	2.3±0.3	6±1
2′‐deoxyuridine	n.d.	n.d.
2′‐deoxycytidine	n.d.	n.d.

[a] Reactions were carried out at 5 mm scale in 100 mm phosphate buffer pH 7.5 at 37 °C with 2 units of HePNP. [b] n.d.=not detected.

The lower *K*_m_ value (1.03 mm) of HePNP for inosine compared to other PNPs[^24^, ^25^] and the lower cost of the substrate^[26]^ led to the selection of inosine as the sugar donor for further transglycosylation reactions (Figure S6). Then, the influence of the enzyme concentration and the phosphate buffer on the phosphorolysis reaction were investigated. As previously reported, the amount of enzyme does not affect the equilibrium, which appears to be limited to 50 % m.c. with 2, 4, or 10 units of HePNP (Table S1A).^[19]^ On the contrary, the concentration of phosphate buffer can shift the equilibrium towards the phosphorolysis direction (Table S1B).^[19]^

### Immobilization of HePNP: reversible, fast, and strong strategy through a (6 x)Cys‐tag and a thiol‐activated agarose

**Figure 1:**
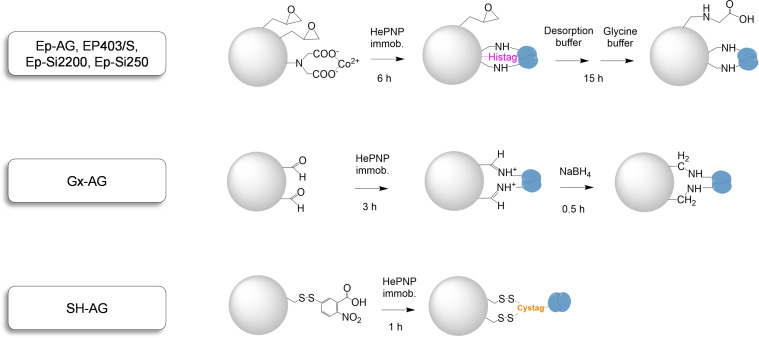
Enzyme immobilization schemes. Ep−AG: epoxy−agarose, EP403/S: epoxy−methacrylate; Ep−Si2200 and EpSi250: epoxy−silica; Gx−AG: epoxy−agarose; and SH−AG: thiol−agarose.

To increase the stability of the biocatalyst as well as to allow its recyclability, the immobilization of HePNP on different supports by using three binding chemistries was performed (Figure 1). Epoxy‐activated supports containing cobalt chelates to drive the (6x)His‐tag‐enzyme were used for the covalent and irreversible immobilization of HePNP on agarose microbeads (Ep−AG), methacrylate microbeads (EP403/S), and silica microbeads (Ep−Si2200 and Ep−Si250).^[27]^ Covalent irreversible immobilization was also achieved on aldehyde activated agarose microbeads (Gx−AG).^[28]^ In all cases more than 90 % of enzyme was covalently immobilized achieving an activity of 3.6–7 U g^−1^ on the support (Tables 2 and S7). The irreversible binding to Ep−Si2200, Ep−Si250, Gx−AG, Ep−AG, and EP403/S was confirmed by SDS‐PAGE (Figure S7). The protein loading was also optimized, obtaining a biocatalyst with 5 mg g^−1^ as the most convenient option (Figure S8).

**Table 2:** Immobilization parameters obtained for the different supports and chemistries.^[a]^

Material support	Reactive group	Matrix	Immobilization yield^[b]^ [%]	Recovered activity^[c]^ [%]	Immobilized activity^[d]^ [U g^−1^_support_]
Ep−AG	epoxy/Co^2+^	agarose	93	38	7.0
EP403/S	epoxy/Co^2+^	methacrylate	97	26	5.0
Ep−Si2200	epoxy/Co^2+^	silica	100	18	4.0
Ep−Si250	epoxy/Co^2+^	silica	99	20	3.6
Gx−AG	aldehyde	agarose	99	29	5.7
SH−AG	thiol	agarose	94	37	6.9

[a] The protein offered was 1 mg g^−1^. Ep−AG: epoxy−agarose, EP403/S: epoxy−methacrylate; Ep−Si2200 and EpSi250: epoxy−silica; Gx−AG: epoxy−agarose; and SH−AG: thiol−agarose. [b] Immobilization yield was calculated as: (protein in solution−protein in the supernatant after immobilization)/protein in solution ×100. [c] Recovered activity: specific activity of the free enzyme/specific activity of the immobilized enzyme ×100. [d] Immobilized activity on the support: activity of the immobilized enzyme/g of support.

Nevertheless, irreversible binding chemistries hamper the recycle and reuse of the expensive supports following inactivation of the enzyme over time. As an alternative to the irreversible covalent immobilization, the enzyme was immobilized on agarose microbeads activated with thiol groups (SH−AG) through disulfide bonds harnessing the (6x)Cys‐tag (Figure S7).

This faster and simpler immobilization strategy was previously developed but never applied to enzyme immobilization for biocatalytic applications (Figure 1).^[29]^ Unlike weaker reversible immobilization strategies (i. e., ionic interactions), the disulfide bonds keep the enzyme covalently bound to SH−AG avoiding lixiviation of the enzyme during the reaction while it can be selectively reversed, under precise chemical conditions. With low concentrations of DTT m(dithiothreitol) (0.1–5 mm), a requirement for some enzymes, the immobilized HePNP on SH−AG remained attached to the support (Figure S9), while when a harsher DTT treatment was applied (50 mm), the enzyme could be efficiently cleaved from the AG−SH support, enabling its recycling for the immobilization of fresh enzyme (Figures S7 and S10).

Besides, this strategy, while providing several attachment points through the residues of the (6x)Cystag, allows also the oriented and selective immobilization of HePNP on the support exclusively on the (6x)Cys‐tag, rather than random multi‐point attachment via the nucleophilic groups on the protein surface and the epoxy or aldehyde moieties on the supports that may lead to structure distortion and loss of enzyme activity. Indeed, cysteine‐based tags have been previously applied for selective binding and labelling of proteins due to the typical absence of cysteine residues on the external surface of proteins.[^30^, ^31^] The structure analysis performed with CapiPy^[32]^ confirmed that no exposed cysteines are present on the protein surface of HePNP, thus the immobilization happens specifically by the (6x)Cys‐tag (Figure S11). The resulting immobilized HePNP on SH−AG showed to be as active as the enzyme irreversible immobilized on Ep−AG, and more active than HePNP immobilized in any of the other materials (with any binding chemistry) tested (Table 2).

### High co‐solvent and thermal stability of HePNP

In order to develop an effective biocatalyst, the enzyme must maintain its activity as long as possible and be resistant to the required reaction conditions. The free HePNP showed a remarkable stability, retaining more than 80 % activity after storage at room temperature for 32 days (Figure S12A).

Specifically for the biosynthesis of nucleoside analogues, the stability in the presence of co‐solvents is highly relevant owed to the poor water‐solubility of the nucleosides and nucleobases. Dimethyl formamide (DMF) has been previously used as co‐solvent for the synthesis on nucleoside analogues with a high stability of the immobilized PNP from *A. hydrophila*.[^8^, ^11^] However, the significant toxicity and hazardousness of DMF discouraged its use for a sustainable synthetic approach. Encouraged by the good activity results obtained with DMSO (Figure S5D) and the good solubility of the inosine, we tested the stability of the free and immobilized HePNP on Ep−AG in DMSO. The free enzyme preserved 50 % of the activity after 3 days in presence of 50 % DMSO (Figure S13), while the immobilized version maintained more than 90 % under the same conditions. Moreover, the stability of the immobilized enzyme did not change among the different supports used (Figure S14A). Even for enzymes cloned from *H. elongata*, this stability against high DMSO concentrations would not be common as no more than 50 % of the enzyme activity would be preserved after 24 h of incubation with 20 % DMSO in the best case.[^20^, ^22^, ^33^] Therefore, these results certainly encourage the use of HePNP for biocatalytic applications when the addition of co‐solvent is needed.

In addition to the use of solvents, high temperatures have been described to positively affect the phosphorolysis yields.^[19]^ Again, the high temperature stability of HePNP (90 % of activity after 24 h and 60 % of activity after 4 days at 45 °C) is another advantage of this biocatalyst (Figure S12B). Thermostable enzymes certainly have a higher longevity, which is beneficial to achieve a longer operational stability under continuous flow processes. Such stability is comparable to the PNP from *A. migulanus* and very superior to the PNP from *B. stearothermophilus*.[^25^, ^34^] The temperature stability was further improved after immobilization (Figure S12C).

### (Flow) synthesis of pharmaceutically relevant nucleoside analogues

Immobilized HePNP on SH−AG was employed as a catalyst for the phosphorolysis of inosine and transglycosylation reactions as a one‐pot one‐enzyme approach (Scheme 1). To maximize the production of purine nucleoside analogues, the effect of the phosphate and the nucleobase (6‐*O*‐methylguanine) (**1**) concentration on both reaction steps was studied. An improvement on the phosphorolysis step was noted when the phosphate concentration was increased up to 500 mm (Table S2 and Figure S15A) but, as expected, it decreased the efficiency of transglycosylation due to the shift of the equilibrium towards the phosphorolysis direction (Scheme 1). Indeed, we observed that the equilibrium of the batch reactions was reached in all cases after 2 h, and the supplementation of the reaction with additional enzyme did not lead to a higher conversion (Figure S15). The phosphate concentration was therefore set at 20 mm, which showed the best transglycosylation yield (Table S2). Regarding the sugar donor (inosine), other works, however, stated in general the use of 1–4 equivalents of different sugar donors, with yields spanning greatly from low percent to high eighties, with the highest reported the synthesis of adenosine using uridine.[^7^, ^9^, ^11^, ^25^, ^35^] Our results are indeed in agreement, and as high as 20 equivalents (100 mm) of inosine were required to observe total conversion at 5 mm scale in batch conditions and after 24 h (Table S3).

The continuous synthetic process was then investigated. Immobilized HePNP on SH−AG (4 mg g^−1^; 18.6 U g^−1^) was loaded into a packed‐bed reactor (PBR) connected to a continuous flow system (Table 3) and its performance assayed for the synthesis of a Nelarabine analogue, obtaining the best flow‐reaction conditions with just 2 min residence time (R.T.) and 37 °C (Table S4). Similarly to batch mode, high conversion yields (80–90 %) were obtained when using 5 equiv. of sugar donor at 5 mm scale in a shorter period of time.

**Table 3:** Biosynthesis of nucleoside analogues by the immobilized HePNP.^[a]^

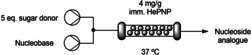					
Entry	Nucleobase	Transglycosylation [%]			
		batch (24 h)	flow (2 min)	flow (10 min)	flow (30 min)
1	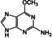	80±2.1	56±1.2	91±2.1	n.t.
2	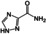	29±5.4	7±2.3	15±3.2	14±3.1
3	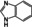	30±2.6	6.9±1.6	12±3.4	21±0.6
4	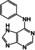	65±0.1	2.4±0.2	17±2.8	54±2.8
5	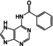	56±2.9	1.9±0.1	14±1.8	19±1.1

[a] The reactions were performed with 25 mm inosine and 5 mm nucleobase in 20 mm phosphate buffer at pH 7.5 and 37 °C. Batch reactions were carried out in 2 mL with 2 U of enzyme. For the flow reactions, the volume of the PBR (4 mg g^−1^ immobilized enzyme) was 1.7 mL. Retention times are depicted. n.t.=not tested.

With those conditions, five nucleobases [6‐*O*‐methylguanine (**1**), 1,2,4‐triazole‐3‐carboxamide (**2**), benzimidazole (**3**), anilinepurine (**4**), and benzamidepurine (**5**)] were investigated to achieve the corresponding nucleoside analogues (Table 3). A Nelarabine analogue, a very valuable orphan drug chemotherapeutic, was synthesized using **1** as a nucleobase and yielding >90 % of conversion in 10 min, in agreement with previous reports.^[18]^ Then, we tested the synthesis of the antiviral drug Rivabirin that is utilized for treatment of Hepatitis C and COVID‐19, which are currently two of the most important health problems worldwide.^[36]^ By using the nucleobase **2**, 29 % of conversion was obtained in batch reactions and a maximum of 14 % of conversion in flow. While these yields are moderate, it must be noted that the one‐pot one‐enzyme biosynthesis of Ribavirin, using purified PNP, has been attempted also by others obtaining even poorer productivities or no activity at all.[^7^, ^24^]

Other approaches such as the combination of two purified enzymes (PNP and UP), whole cells, or chemo‐enzymatic strategies showed to be more promising.[^6^, ^25^] The substrate scope of HePNP was tested further with a simple bezimidazole and also with bulky nucleobases such as **4** and **5**. The resulting nucleosides of **4** and **5** are patented as pharmaceuticals for cancer treatment.^[37]^ Such enzymatic syntheses have not been described to date. Interestingly, HePNP accepted both bulky substrates giving satisfactory transglycosylation yields when the biotransformations were performed in batch for 24 h. Yet, these reactions were quite dependent on the residence time when implemented in flow conditions, achieving better yields with longer R.T. Unexpectedly, despite the similar bulky structures of **4** and **5**, better results were obtained with **4** compared to **5**, specially under flow conditions. Docking analysis of both resulting nucleosides (N6‐phenyl‐adenosine and N6‐benzoyl‐adensoine) were carried out to try and rationalize those differences. The N7 of the nucleobase ring was found to be at the same distance from the catalytic residue (Asp205) as the N7 of the inosine (Figure S16) and the active site of HePNP is sufficiently spacious to accommodate both bulky substrates (Figure S17A–C). However, the aromatic group of **4** can be stabilized by Phe160 due to a greater proximity compared to the aromatic group of **5** (Figure S17D). In addition, different reaction conditions were tested for the phosphorolysis and transglycosylations using **4** and **5** as nucleobases with the free HePNP. Table S5 shows that neither the concentration of sugar donor nor the concentration of phosphate improves the formation of the desired nucleoside analogue, while a higher concentration of phosphate is indeed counterproductive as shown for **1** (Table S2). However, an increase in the temperature results in high reaction yields. In all cases, the yields obtained using the nucleobase **5** were lower than with **4** (Table S5).

### Intensification of the synthesis of the Nelarabine analogue: flow production, in‐line recycling of sugar donor, and reuse of the support for enzyme immobilization

As a proof of concept, the flow synthesis of the Nelarabine analogue was scaled up from 5 to 50 mm testing different concentrations of **1** to find the optimal nucleobase concentration to develop an efficient synthetic process. The conversion [%] at 5 mm was slightly better than at 10 mm scale, while at 25 and 50 mm the conversions decreased more dramatically when using 2 equiv. of inosine (Figure S18). Thus, 10 mm was chosen for this experiment. As shown in the Table S4, 18 equiv. of inosine was required to achieve the total conversion of the Nelarabine analogue. With those conditions (10 mm **1**, 180 mm inosine), the biosynthesis was implemented in flow sustaining >99 % of conversion during 100 column volumes (Figure S19 and Table 4, step 1). The calculated space‐time yield for this system is 89.1 g L^−1^ h^−1^ with a catalyst productivity of 6.1 mmol_product_ mg^−1^_enzyme_ in 24 h which are the highest metrics reported so far not only for the flow synthesis of Nelarabine analogue but also for the flow synthesis of any nucleoside analogue.[^9^, ^11^] The sustainability of the system was also assessed by calculating the E‐factor, which was 14.6 (Table S6). This value is at the low end with respect to other synthesis of nucleoside analogues previously reported (10–30).^[38]^

**Table 4:** Flow process intensification of the synthesis of the Nelarabine analogue at 10 mm scale with in‐line recycle of the sugar donor during 20 cycles.^[a]^

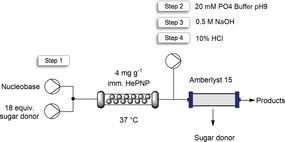			
Separation of sugar donor in step 1	Separation of sugar donor in step 2	Recovery of products	
		Nelarabine analogue	Hypoxantine
84 %	16 %	78 %	92 %
200 mg	38 mg	23 mg	17.4 mg

[a] The biotransformation by the immobilized HePNP in a PBR was performed at 37 °C with R.T. 2 min and a flow‐rate of 0.85 mL min^−1^. Step 1 refers to the biotransformation in the PBR and the first separation of sugar donor. For steps 2–4, the solution in the inlet was changed depending on the desired process.

While the sugar donor is the cheaper reagent in the set‐up, clearly such an excess lead to major waste on a large scale. In order to make the system more efficient, the excess of sugar donor was recovered by an in‐line catch‐and‐release strategy based on previous works developed in our group.[^20^, ^39^] A single column packed with the resin Amberlyst® A15 was utilized to trap the product (Nelarabine analogue) and the by‐product of the reaction (hypoxanthine) while 84 % of the inosine was flushed out (Table 4, step 1). Amberlyst® A15 is an acidic cation exchange resin that can bind to basic molecules such as nucleobases and nucleoside analogues. Since the sugar donor is in excess, this is flushed out once the column is saturated. The inosine was recirculated into the system for its reuse in a new flow reaction obtaining similar conversions. Following these steps, the E‐factor was further reduced from 14.6 to 4.2, as the buffer and most of the sugar donor could be readily recycled (Table S6). Then, a washing step with buffer at pH 9.0 removed any remaining trace of inosine which may have a lower affinity for the A15 column (Table 4, step 2). Finally, the products trapped in the column were released by adding an inlet of 0.5 m NaOH (Table 4, step 3), and the A15 column was easily and quickly regenerated with 10 % HCl (Table 4, step 4).

The immobilized biocatalyst on SH−AG still retained 50 % of activity after 2 months of usage for reactions in flow and storage at 4 °C when it was not in use (Figure S20). Typically, the whole immobilized biocatalyst, once its activity falls below an acceptable threshold, is replaced by a fresh immobilized biocatalyst, even when in theory the spent enzyme could be displaced. Although in this case the immobilized enzyme has a remarkable stability, commercial agarose‐based supports are still very expensive (1000–2000 € kg^−1^),^[40]^ reducing the cost‐efficiency of the process and discouraging its implementation at industrial scale. Our immobilization strategy exploited a reversible covalent bonding (Figure S10), which we selected to test the reusability of the support by cleaving the inactive enzyme and the re‐immobilization of a fresh enzyme solution directly in the flow setup.

This increases the automation of the process, avoiding the time‐consuming steps of disassembling the PBR, displacing of the enzyme in batch, re‐immobilization in batch, and re‐assembling of the PBR. The successful immobilization resulted in 90 % of immobilization yield (3.6 mg g^−1^), obtaining a biocatalyst with 19 % of recovered activity and 13.7 U g^−1^ which very well compares with the biocatalyst obtained under the same conditions in the first immobilization round (Figure S20).

## Conclusion

Despite the great progress on enzymatic synthesis of nucleoside analogues in the last years, greener and more efficient strategies are demanded for the implementation at industrial scale. Moreover, a broader substrate scope of the nucleoside phosphorylases is highly desired, especially for large substrate molecules. In this work, we have addressed the key points for its optimization: a novel and highly stable enzyme; a reversible, strong, and quick strategy for enzyme immobilization; the process intensification in flow reaching total conversion by using a simple and portable system such as 1.7 mL reactor; the biocatalytic synthesis of bulky nucleosides never done before; and the recycling of the excess of sugar donor and waters into the system. The overall waste production has been significantly minimized by recycling not only the immobilized enzyme but also the high‐cost material support after inactivation of the enzyme. In fact, this immobilization strategy pioneers the reusability of the support microbeads for further enzymatic reactions. As proof of concept, a Nelarabine analogue which is a very valuable nucleoside analogue (7920 € g^−1^) has been produced by using cheaper starting materials (sugar donor+nucleobase=571.5 € g^−1^).^[26]^

## Experimental Section

### Materials

All chemical reagents were acquired from Sigma Aldrich (Gillingham, UK), Thermo Fisher (Loughborough, UK), Acros Organics (Reinach, Switzerland), or Fluorochem (Hadfield, UK) and used without further purification. Plain agarose (6BCL) was purchased from Agarose Bead Technologies (Madrid, Spain). The methacrylate supports were purchased from Resindion S.R.L (Milan, Italy). Sipernat® 2200‐PC and Sipernat® 250‐PC were kindly donated Evonik (Essen, Germany). Amberlyst® A15 was acquired from Acros Organics.

### HePNP expression, purification, and quantification

The plasmid pET28b harboring the gene of HePNP (Figure S1) was transformed into E. coli BL21(DE3) by heat‐shock at 42 °C. Then, 1 L flasks containing 300 mL of LB with 100 μg mL^−1^ kanamycin were inoculated with 3 mL of an overnight culture and incubated at 37 °C and 150 rpm until OD600 was 0.6–0.8. To induce the protein expression, different conditions were trialed (Figure S2A) finding the best HePNP expression with the addition of 1 mm IPTG and incubation at 21 °C overnight. Cells (2 g) were harvested by centrifugation at 4500 rpm and resuspended in 6 mL of 50 mm phosphate buffer, 100 mm NaCl, and 30 mm imidazole at pH 8.0. The suspension was placed on ice and sonicated at 40 % amplitude for 10 min, with pulses of 5 s ON, 10 s OFF. After centrifugation at 14500 rpm for 45 min, the supernatant was filtered with a 0.45 μm filter and HePNP was purified from the supernatant by a Ni−NTA column in the AKTA‐pure FPLC. HePNP was eluted in 50 mm phosphate buffer, 100 mm NaCl, and 300 mm imidazole at pH 8.0. The purified enzyme was dialyzed twice in 100 mm phosphate buffer at pH 7.5 containing 10 % glycerol. The protein concentration was estimated by measuring the absorbance at 280 nm in the EPOCH2 (nanodrop Tek3 plate) and using the predicted extinction coefficient 17920 m cm^−1^ and the molecular weight of 31981 Da obtained from https://web.expasy.org/protparam. Low protein concentrations (<1 mg mL^−1^) were determined by Bradford assay by measuring the absorbance at 595 nm of a solution containing 5 μL of protein sample with 250 μL of Bradford reagent. A standard curve was done with the protein BSA.

### Activity assay

The activity of HePNP (5 μg mL^−1^ of free enzyme; 50 mg of immobilized enzyme) was tested with 5 mm inosine in 100 mm potassium phosphate buffer at pH 7.5 in a reaction volume of 5 mL. The reactions were incubated at 37 °C under shaking and monitored after 2, 4, 6, 8, and 10 min by withdrawing 100 μL of the reaction mix and adding 200 μL of acetonitrile and 200 μL of 0.2 % HCl in H_2_O. The samples were filtered with a 0.45 μm PTFE filter prior HPLC analysis. The specific enzyme activity (U mg^−1^) was calculated as following the Equation 1:

(1) $$Activity=\frac{\frac{substrate\;conc.\;\left[mM\right]\times conv.\;\left[\%\right]}{t\;\left[min\right]}\times V\;\left[mL\right]}{enzyme\;\left[mg\right]}$$

The conversion [%] was calculated from the HPLC analyses of the reaction mixtures following the Equation 2:

(2) $$Conv.=\frac{product\;area}{product\;area+substrate\;area}\times 100\;\%$$

The molar conversions [%] were also confirmed by using standard curves of the substrates and products (1–50 mm).

### Enzyme biotransformations in batch mode

Batch reactions with soluble enzyme (2 units) were incubated at 37 °C and under shaking for 24 h in 2 mL of reaction mix containing 5 mm of sugar donor and 10 mm of nucleobase in 100 mm phosphate buffer at pH 7.5, unless otherwise specified. Batch reactions with immobilized enzyme were triggered adding 50 mg of 5 mg g^−1^ immobilized HePNP, unless otherwise specified. The reactions were monitored by HPLC.

### Activation of supports and enzyme immobilization

**Ep−AG (agarose microbeads activated with epoxy groups)**: Epoxy−agarose was prepared as previously described.^[27]^ The addition of Co^2+^‐chelates was carried out by incubating 1 g of epoxy−agarose with 2 mL of modification buffer (0.1 m sodium borate, 2 m iminodiacetic acid, pH 8.0) for 2 h. After filtration and washing, the support was incubated with 5 mL of Metal Buffer (30 mg mL^−1^ of CoCl_2_). After filtration and washing, 5 mL of enzyme solution were added to the Ep/Co^2+^‐AG and the suspension was incubated for 6 h under shaking. The immobilized enzyme was washed with 3 mL of desorption buffer (50 mm EDTA, 0.5 m NaCl in 50 mm phosphate buffer, pH 7.2). Finally, the remaining epoxy groups were blocked by incubation with 4 mL of 3 m glycine, pH 8.5 overnight and afterwards the immobilized enzyme was washed with 100 mm phosphate buffer at pH 7.5 before its storage.

**EP403/S (methacrylate microbeads activated with epoxy groups)**: EP403/S activated with epoxy groups was commercially available. The addition of Co^2+^‐chelates and the enzyme immobilization were carried out as described in the previous section for Ep−AG.

**Ep−Si2200 and Ep−Si250 (silica microbeads activated with epoxy groups)**: 1 g of silica microbeads (Sipernat® 2200‐PC and Sipernat® 250‐PC) was washed and resuspended in 2 mL of H_2_O. Then, 0.5 mL of 25 % of 3‐glycidyloxypropyl trimethoxysilane in acetone was added drop by drop to the previous suspension. The suspension was incubated overnight at 25 °C under shaking. After filtration and washing, the addition of Co^2+^‐chelates and the enzyme immobilization were carried out as described in the previous section for Ep−AG.

**Gx−AG (agarose microbeads activated with aldehyde groups)**: Epoxy−agarose was activated with glyoxyl groups following a modified version of a previous protocol.^[28]^ Briefly, 1 g of epoxy−agarose was incubated with 10 mL of 100 mm H_2_SO_4_ overnight under orbital shaking. Then, the support was filtered and washed. The resulting glyceryl support was oxidized with 10 mL of 30 mm NaIO_4_ for 2 h under orbital shaking. After filtration and washing, 10 mL of enzyme solution in 100 mm sodium bicarbonate buffer at pH 10.0 were mixed with 1 g of AG−Gx and incubated under orbital shaking for 3 h. Then, the suspension was filtered and incubated with 10 mL of 1 mg mL^−1^ NaBH_4_ for 30 min to reduce the imine bonds.

**SH−AG (thiol−agarose)**: The activation of SH−AG was based on a previously reported protocol.^[29]^ Briefly, 1 g of epoxy−agarose was incubated with 10 mL of 50 mm Na_2_S in 100 bicarbonate buffer at pH 10 and incubated overnight under shaking. After filtration and washing, 10 mL of 10 mm DTNB (2,2′‐dinitro‐5,5′‐thiobenzoic acid) were added to the agarose and the suspension was incubated for 3 h. After filtration and washing thoroughly, 4 mL of the enzyme solution were added and incubated for 1–2 h under shaking.

The immobilization yields [%] were calculated as: (protein in solution−protein in the supernatant after immobilization)/protein in solution ×100. The protein concentration in the offered solution and the supernatant after immobilization was determined by Bradford assay. The activity of the offered protein and the remained activity in the supernatant after immobilization was also tested. The immobilized enzymes were washed with 100 mm phosphate buffer at pH 7.5 for its storage at 4 °C.

### Stability tests

The enzyme (0.5 mg mL^−1^ of free HePNP; 100 mg of immobilized HePNP) was incubated in 1 mL of 100 mm phosphate buffer at pH 7.5. At different time points, 100 μL of enzyme solution/suspension were withdrawn to measure the activity as described above.

**Co‐solvent stability**: The enzyme solution/suspension was incubated at 25 °C for 72 h.

**Temperature stability**: The enzyme solution/suspension was incubated at 30, 37, or 45 °C for 72 h.

**Stability in presence of DTT**: 50 mg of immobilized enzyme (1 mg g^−1^) were incubated with 0.5 mL of DTT at different concentrations for 24 h at 25 °C.

### Flow reactions

Flow biotransformations were performed using a R2S/R4 Vapourtec flow reactor equipped with a V3 pump and an Omnifit glass column (6.6 mm i.d. ×100 mm length) filled with the immobilized enzyme (1–2 g with 4 mg g^−1^ of protein loading) as a packed‐bed reactor (PBR). A first equilibration step was performed by running 100 mm phosphate buffer pH 7.5 buffer at 0.5 mL min^−1^ for 10 min. Then, the solutions of substrates at different concentrations were mixed in a T‐tube and pumped through the PBR containing the immobilized biocatalyst. The flow‐rate was adapted depending on the desired residence time for each reaction. Samples were collected after each column volume and analyzed by HPLC.

### Calculation of E‐factor

The E‐factor was calculated according to the Equation 3:^[41]^

(3) $$E-\mathrm{factor}=\frac{total\;mass\;of\;waste}{mass\;of\;final\;product}$$

To this end, the mass [μmol] of each reagent (inosine, 6‐*O*‐methylguanine, hypoxantine, Nelarabine analogue, phosphate, water) used in the flow reaction was estimated. Both the phosphorolysis and the transglycosylation conversions [%] during 100 column volumes were considered for the calculations. Therefore, the E factor was calculated as the sum of the mass all the reagents that could not be reused during the flow reaction (inosine, 6‐*O*‐methylguanine, hypoxantine, phosphate, water) divided by the mass of the Nelarabine analogue produced. Then, the E‐factor after the addition of the Amberlyst® A15 column was calculated as the sum of the inosine that could not be recycled, 6‐*O*‐methylguanine, and hypoxantine, divided by the Nelarabine analogue produced, since the buffer and 84 % of the inosine could be reused (Table S6).

### In‐line product separation and recovery of sugar donor

A second column with 2.5 grams of Amberlyst® A15, which is a strongly sulfonic acid resin for ion exchange, was connected in‐line after the PBR with the immobilized HePNP. The A15 column was firstly washed with milliQ H_2_O for 5 min at 1 mL min^−1^ and equilibrated with 50 mm phosphate buffer pH 7.5 for 5 min at 1 mL min^−1^. After running the product of the reaction, a washing step was performed with 20 mm phosphate buffer (pH 9.0) for 10 min at 0.5 mL min^−1^. The final desorption was accomplished by flushing 0.5 m NaOH for 10 min at 0.5 mL min^−1^. Ultimately, the A15 resin was washed with milliQ H_2_O for 10 min at 1 mL min^−1^ and regenerated by running 10 % HCl for 10 min at 0.5 mL min^−1^.

### In flow desorption and re‐immobilization of HePNP

A solution of 50 mm DTT at pH 6.5 was flushed through the PBR at 0.5 mL min^−1^ for 10 min. Then, a washing step was performed by running 50 mm phosphate buffer at pH 7.5 at 1 mL min^−1^ for 5 min. The agarose support was reactivated by running a column volume of 50 mm DTNB at pH 8.5. The flow was stopped, and the agarose support was incubated for 1 h with the DTNB solution. Afterwards, an enzyme solution was passed through the column at 0.2 mL min^−1^ and the flow was stopped again to incubate the enzyme with the SH−AG for 1 h and allow the enzyme immobilization (protein offered: 4 mg g^−1^). The immobilization yield was calculated by assaying the enzyme activity in the flow‐through of the PBR. Finally, the PBR was washed with 50 mm phosphate buffer at pH 7.5. To determine the recovered activity [%] after immobilization and the activity on the SH−AG [U g^−1^], the reactor was fed with a solution of 5 mm inosine in 100 mm phosphate buffer at pH 7.5 and the product was compared to the results obtained with the same reaction conditions by using the previous the previous immobilized enzyme.

### HPLC analysis

The products were analyzed by HPLC (Dionex UltiMate 3000 (Thermo Fisher, Loughborough, UK), equipped with a C18 column 3.5 μm, 2.1×100 mm (Waters, Elstree, UK). 2 μL of sample were injected and submitted to a gradient method 5 : 95 to 95 : 5 [H_2_O/ACN containing 0.1 % trifluoroacetic acid (TFA)] over 4 min with a flow rate of 0.8 mL min^−1^ at 45 °C. For the analysis of the ribavirin synthesis, a gradient method 1 : 55 to 55 : 1 (H_2_O/:MeOH) over 8 min was used. The samples were detected using UV detectors at the following wavelengths: 250 nm (inosine 2.4 min; hypoxantine 2.25 min; deoxyinosine 2.25 min; guanosine 2.7 min; triazole carboxamide 2.12 min; rivabirin 2.24 min), 265 nm (adenosine 2.75 min; deoxyadenosine 2.4 min; thymidine 4 min; uridine 2.38 min; deoxyuridine 2.78 min; deoxycytidine 2.4 min; benzimidizole 4.34 min) and 280 nm (6‐*O*‐methylguanosine 3.9 min; nelarabine analogue 4.2 min; anilinepurine 4.8 min; N6‐phenyl‐adenosine 4.6 min; benzamidepurine 4.8 min; N6‐benzoyl‐adenosine 4.6 min).

## Conflict of interest

The authors declare no conflict of interest.

## Supporting information

As a service to our authors and readers, this journal provides supporting information supplied by the authors. Such materials are peer reviewed and may be re‐organized for online delivery, but are not copy‐edited or typeset. Technical support issues arising from supporting information (other than missing files) should be addressed to the authors.

Supporting Information
