# Supplementary material for: Sustainable Flow‐Synthesis of (Bulky) Nucleoside Drugs by a Novel and Highly Stable Nucleoside Phosphorylase Immobilized on Reusable Supports
Source: ChemSusChem. 2021 Nov 27;15(1):e202102030. doi: 10.1002/cssc.202102030 (PMC9298701; doi:10.1002/cssc.202102030)
Supplement: Supplementary file 1 — Supporting Information [file CSSC-15-0-s001.pdf]

ChemSusChem

Supporting Information

Sustainable Flow-Synthesis of (Bulky) Nucleoside Drugs by a Novel and Highly Stable Nucleoside Phosphorylase Immobilized on Reusable Supports

Ana I. Benítez-Mateos and Francesca Paradisi*© 2021 The Authors. ChemSusChem published by Wiley-VCH GmbH. This is an open access article under the terms of the Creative Commons Attribution License, which permits use, distribution and reproduction in any medium, provided the original work is properly cited.

Supplementary information

Sustainable flow-synthesis of (bulky)nucleoside drugs by a novel and highly stable nucleoside phosphorylase immobilized on reusable supports

Ana I. Benítez-Mateos[a] and Francesca Paradisi*[a]

[a] Department of Chemistry, Biochemistry and Pharmaceutical Sciences, University of Bern. Freiestrasse 3, 2012 Bern, Switzerland

Content

Figure S1. Plasmid map of pET28b_HePNP and protein sequence 3

Figure S2. SDS-PAGE analysis of the expression and purification of HePNP 4

Figure S3. Multiple sequence alignment of HePNP with AhPNP, YpPNP, and EcPNP by CLUSTAL 4

Figure S4. Structure analysis of HePNP enzyme 5

Figure S5. Optimization of the reaction conditions for the HePNP activity 5

Figure S6. Michaelis-Menten plot of the free HePNP for inosine 6

Table S1. Influence of the enzyme concentration and the phosphate buffer on the phosphorolysis 6

Figure S7. SDS-PAGE analysis of the HePNP immobilization 7

Figure S8. Optimization of the protein loading of HePNP immobilized on Ep-AG 7

Figure S9. Stability of the binding chemistry between the (6x)Cystagged HePNP and the SH-AG 8

Figure S10. Fluorescence microscopy imaging of FITC-labeled HePNP immobilized on SH-AG 8

Figure S11. Analysis of the HePNP structure by CapiPy 9

Figure S12. Temperature and long-term stability of HePNP 10

Figure S13. Stability towards DMSO of free HePNP and immobilized HePNP 10

Figure S14. Stability of the immobilized HePNP on different supports 11

Table S2. Effect of phosphate buffer concentration on the glycosylation efficiency 11

Figure S15. Equilibrium of the batch reactions at different concentrations of phosphate buffer (5, 20 and 500 mM). 12

Table S3. Effect of sugar donor (inosine) concentration on the glycosylation efficiency 12

Table S4. Optimization of the retention time (R.T.), temperature and sugar donor (inosine) concentration of the continuous flow reactions 13

Figure S16. Docking analysis of inosine, N6-phenyl-adenosine, and N6-benzoyl-adensoine 13

Figure S17. Docking analysis of inosine, N6-phenyl-adenosine, N6-benzoyl-adensoine, and superposition of N6-phenyl-adenosine and N6-benzoyl-adensoine 14

Table S5. Optimization of the sugar donor (inosine) concentration, temperature and phosphate concentration for the reactions using N6-phenyl-adenosine and N6-benzoyl-adenosine as nucleobase 14

Figure S18. Optimization of the nucleobase (6-O-methylguanine) concentration in flow 15

Table S6. Raw data of E-factor calculations 15

Figure S19. Operational stability of the immobilized HePNP on Ep-AG under flow conditions 16

Figure S20. Operational and storage stability of the HePNP immobilized on SH-AG 16

Supporting References 17

A

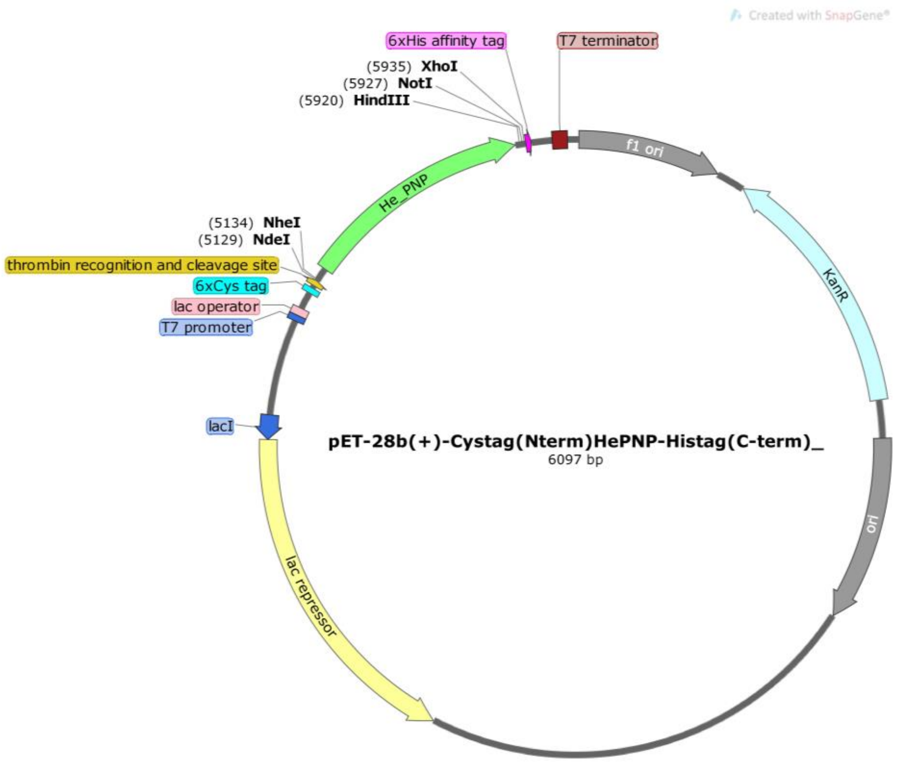

B

Protein sequence:

MGSSCCCCCCSSGLVPRGSHMASMTGGQQMGRDPMATPHIKAERGDFADTVLMPGDPLRARYIAE
TFLDDARLVNEVRNMYGYTGTYRGREISVMGHGMGIPSISIYAKELITEFDVKRLIRVGSCGAVRDDV
AVRDVVIGMGASTDSGVNRTRFLGNDLGAIADFELTRHAVDAAREHGVPVKVGNIFSADLFYDPRPE
MVEMMRRYGIVGVEMEAAGLYGVAAEFGARAATICTVSDHIVKGDSLSSDERATTFDDMMRIALDSV
LRDDAGGDAWNSSSVDKLAAALEHHHHHH

Figure S1. A) Plasmid map of pET28b_HePNP and **B)** protein sequence of the cloned HePNP with the (6x)Cystag in the N-terminal and the (6x)Histag in the C-terminal.

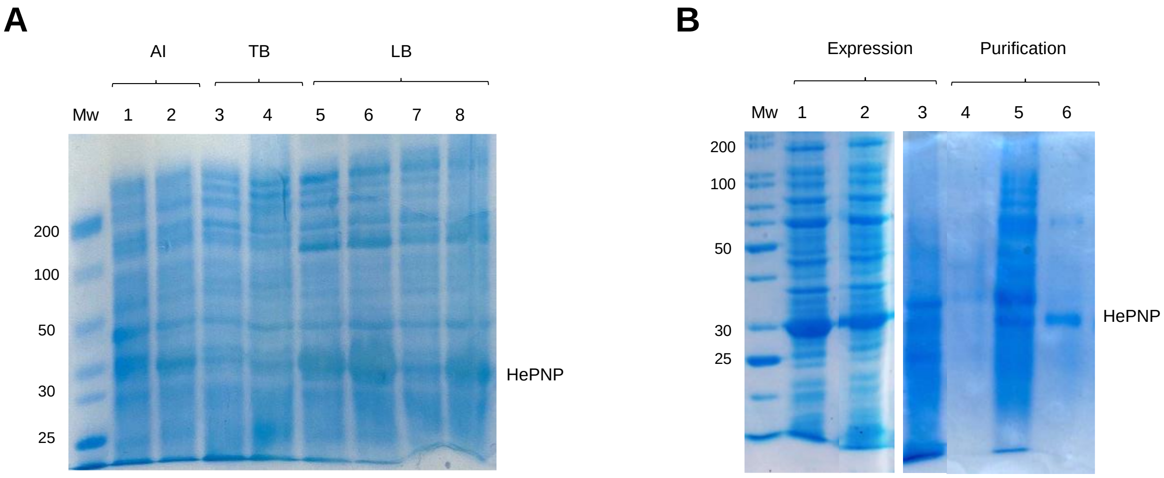

Figure S2. SDS-PAGE analysis of the expression and purification of HePNP (32 kDa). **A)** Optimization of the expression of HePNP in *E. coli.* Line 1: total protein, and line 2: soluble protein fraction after expression in autoinduction medium (AI) at 37 ºC. Line 3: total protein, and line 4: soluble protein fraction after expression in terrific broth (TB) medium at 37 ºC. Line 5: total protein, and line 6: soluble protein fraction after expression in Luria Bertani (LB) medium at 21 ºC. Line 7: total protein, and line 8: soluble protein fraction after expression in Luria Bertani (LB) medium at 37 ºC. **B)** Expression and purification of HePNP. Lines 1: total protein, line 2: soluble protein fraction, and line 3: insoluble protein fraction of the expression in LB at 21 ºC. Line 4: flow-through after washing Line 5: Unbound HePNP. Line 6: purified HePNP Mw: Broad range protein marker from NEB.

```
HePNP      MATPHIKAERGDFADTVLMPGDPLRARYIAETFLDDARLVNEVRNMYGYTGTYRGREISV 60
AhPNP      MATPHINAKDGAFADTVLMPGDPLRAKYIAETFLENVEQVCDVRNMFGFTGTYKGRRISI 60
YpPNP      MATPHINAEMGDFADVVLMPGDPLRAKFIAETFLQDVREVNNVRGMLGFTGTYKGRKISV 60
EcPNP      MATPHINAEMGDFADVVLMPGDPLRAKYIAETFLEDAREVNNVRGMLGFTGTYKGRKISV 60
           ******:*: * ***.**********::******:::.. * :**.* *:****:**.**:

HePNP      MGHGMGIPSISIYAKELITEFDVKRLIRVGSCGAVRDDVAVRDVVIGMGASTDSGVNRTR 120
AhPNP      MGHGMGIPSCSIYAKELITDYGVKTLIRVGSCGAVREDVKLRDVVIGMGACTDSKVNRLR 120
YpPNP      MGHGMGIPSCSIYAKELITDFGVKKIIRVGSCGAVRTDVKLRDVVIGMGACTDSKVNRMR 120
EcPNP      MGHGMGIPSCSIYTKELITDFGVKKIIRVGSCGAVLPHVKLRDVVIGMGACTDSKVNRIR 120
           ********* ***.*****::.** :*********   .* :*********.*** *** *

HePNP      FLGNDLGAIADFELTRHAVDAAREHGVPVKVGNIFSADLFYDPRPEMVEMMRRYGIVGVE 180
AhPNP      FKDHDFAAIADFDLVANAVQAAKNKGVAVRVGNIFSADLFYTPDPSMFDVMEKYGILGVE 180
YpPNP      FKDHDYAAIADFEMTRNAVDAAKAKGVNVRVGNLFSADLFYTPDPQMFDVMEKYGILGVE 180
EcPNP      FKDHDFAAIADFDMVRNAVDAAKALGIDARVGNLFSADLFYSPDGEMFDVMEKYGILGVE 180
           *  .:* .*****::. :**:**:  *: .:***:******* *   .*.::*.:***:***

HePNP      MEAAGLYGVAAEFGARAATICTVSDHIVKGDSLSSDERATTFDDMMRIALDSVLRDDAGG 240
AhPNP      MEAAGIYGVAAEYGAKALTICTVSDHIRTGEQTTSEERQLTFNDMIEIALDSVLLGDN-- 238
YpPNP      MEAAGIYGVAAEFGAKALTICTVSDHIRTGEQTTAAERQTTFNDMIEIALESVLLGDNA- 239
EcPNP      MEAAGIYGVAAEFGAKALTICTVSDHIRTHEQTTAAERQTTFNDMIKIALESVLLGDKE- 239
           *****:******:**:* ********* . :. :: **  **:**:.***:*** .*

HePNP      DA   242
AhPNP      --   238
YpPNP      --   239
EcPNP      --   239
```

Figure S3. Multiple sequence alignment of HePNP with AhPNP (purine nucleoside phosphorylase from *Aeromonas hydrophila*), YpPNP (purine nucleoside phosphorylase from *Yersinia tuberculosis*), and EcPNP (purine nucleoside phosphorylase from *Escherichia coli*) by CLUSTAL O (1.2.4). HePNP shares 70.5% identity with the AhPNP, 70.0% identity with the EcPNP, and 72.6% identity with the YtPNP as analyzed by BLASTX.

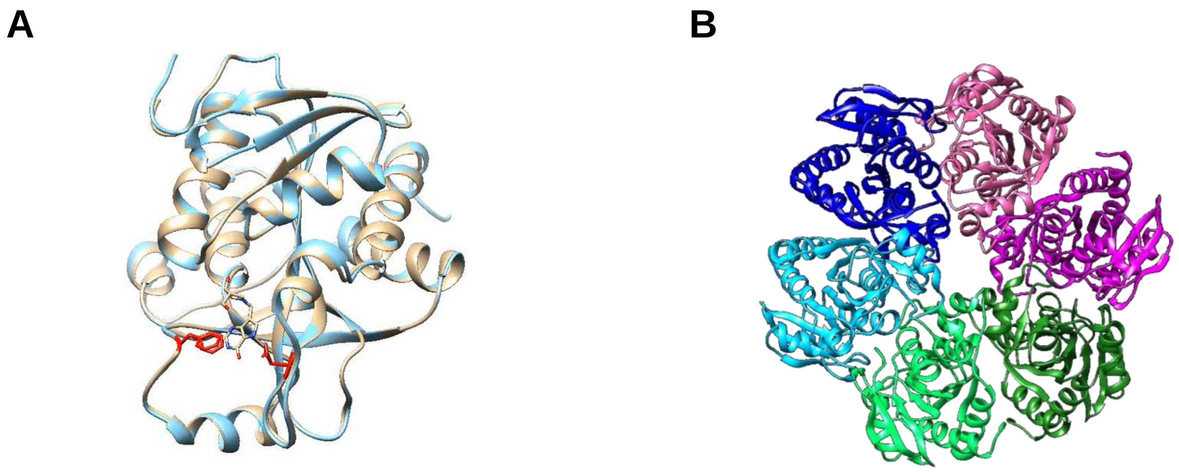

Figure S4. Structure analysis of HePNP enzyme. **A)** Homology model of the HePNP monomer based on the PNP from *Yersinia tuberculosis* (PDB: 3OCC). Inosine is placed in the catalytic pockect. Asp205 and Phe160 are highlighted in red. B) Hexameric structure of HePNP as predicted from the modelling.

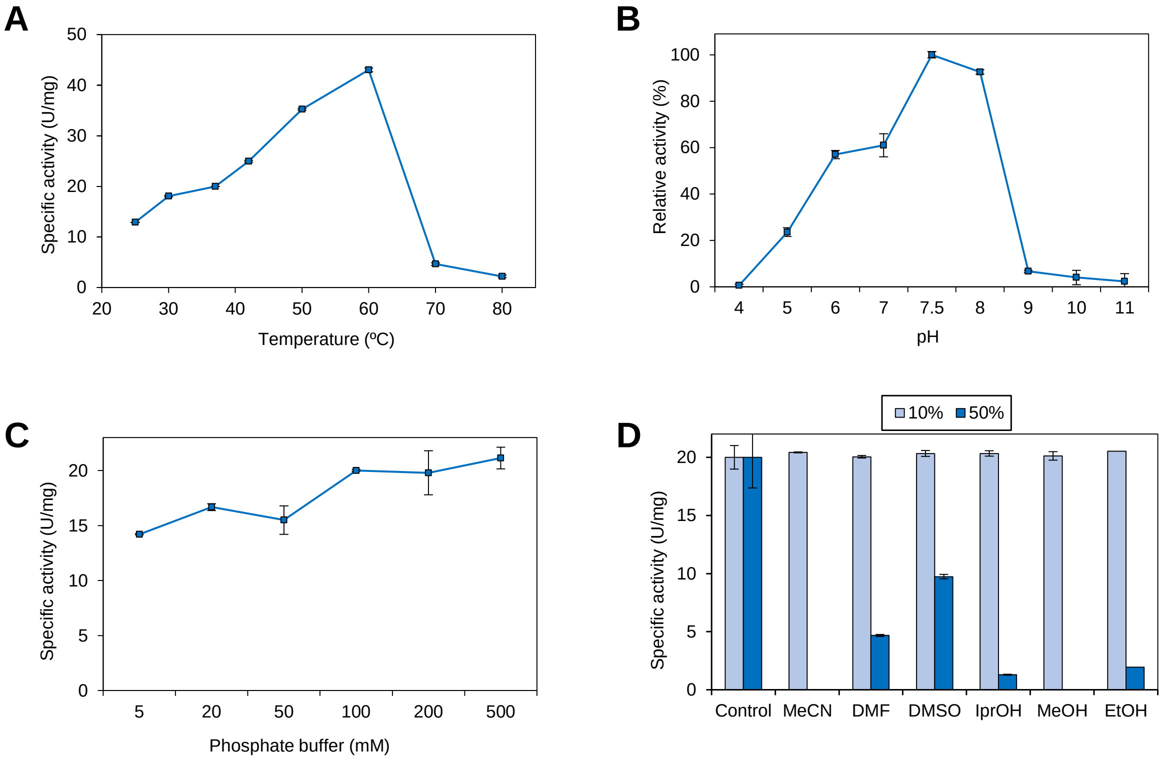

Figure S5. Optimization of the reaction conditions for the HePNP activity. **A)** temperature; **B)** pH (full activity corresponds to 20 U/mg); **C)** ionic strength of phosphate buffer; **D)** presence of co-solvent: acetonitrile (MeCN), dimethylformamide (DMF), dimethylsulfoxide (DMSO), isopropanol (IprOH), methanol (MeOH) and ethanol (EtOH). The reactions were performed with 5 mM inosine and 5 µg/mL HePNP. The standard reactions conditions were 37ºC, 100 mM phosphate buffer at pH 7.5 and 10 minutes of incubation.

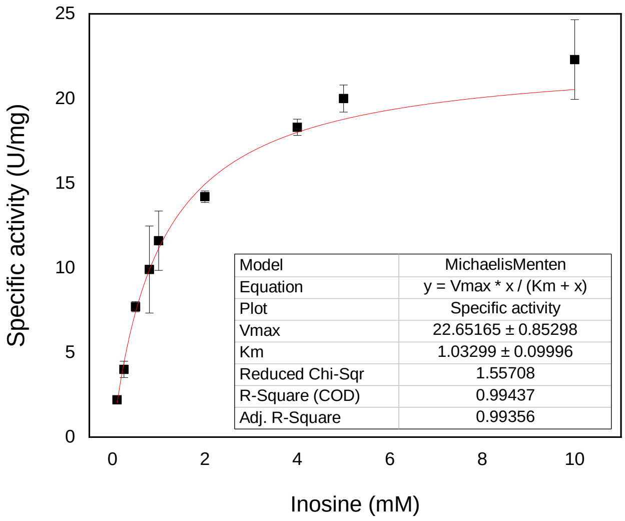

Figure S6. Michaelis-Menten plot of the free HePNP for inosine.

Table S1. Influence of the enzyme concentration and the phosphate buffer on the phosphorolysis reaction. **A)** Monitoring the phosphorolysis of 5 mM inosine in 100 mM phosphate buffer at pH 7.5 at 37ºC for 24 h. **B)** Monitoring the phosphorolysis of inosine with different equivalents of phosphate at pH 7.5 at 37ºC for 24 h. 2 units of HePNP were added to the reactions.

A

HePNP	Phosphorolysis (%)		
	2 h	5 h	24 h
2 U	47.5	51.8	54.8
4 U	49.1	50.6	54.5
10 U	44.8	53.4	56.9

B

Inosine	Phosphate	Phosphorolysis (%)	
		5 h	24 h
5 mM	20 mM (4 eq.)	26.5	25.8
	100 mM (20 eq.)	54.4	59.5
	500 mM (100 eq.)	77.2	78.2
50 mM	100 mM (2 eq.)	22.2	22.4
	500 mM (10 eq.)	41.7	42.8

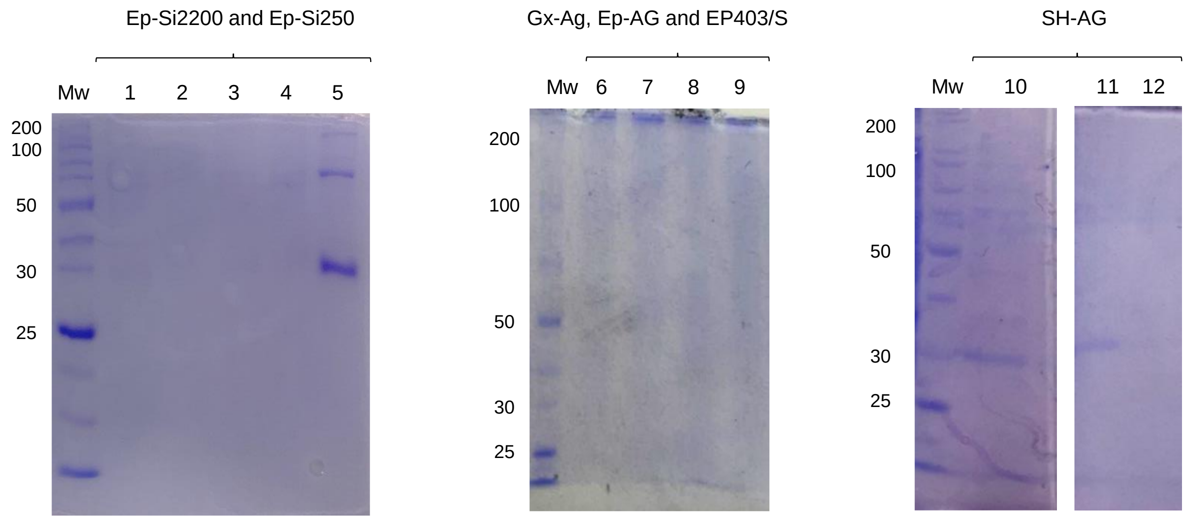

Figure S7. SDS-PAGE analysis of the HePNP immobilization. Line 1: supernatant after immobilization on Ep-Si2200. Line 2: immobilized enzyme on Ep-Si2200. Line 3: supernatant after immobilization on Ep-Si250. Line 4: immobilized enzyme on Ep-Si250. Line 5: free enzyme. Line 6: supernatant after immobilization on Ep-AG. Line 7: immobilized enzyme on Ep-AG. Line 8: immobilized enzyme on Ep-AG at 5 mg/g. Line 9: immobilized enzyme on Ep-AG. Line 10: free enzyme. Line 11: immobilized enzyme on SH-AG. Line 12: supernatant after immobilization on SH-AG. The protein loading was 1 mg/g unless otherwise specified. Mw: Broad range protein marker from NEB.

**U/g**	6.8	18.6	23.3	28.3	48.5

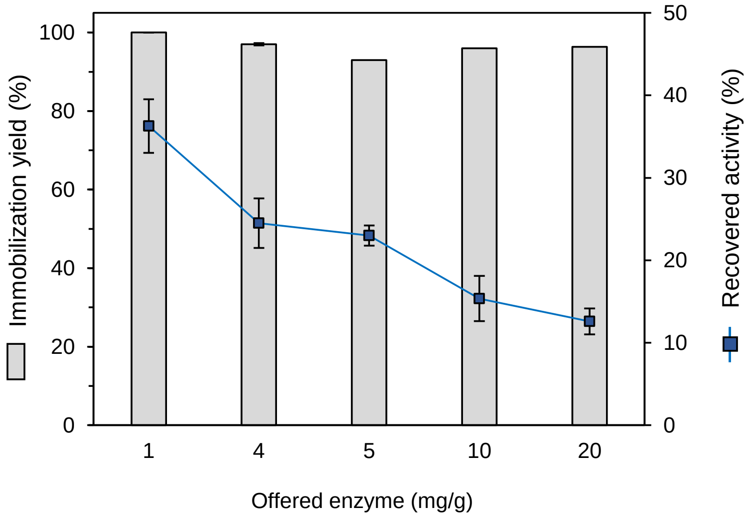

Figure S8. Optimization of the protein loading of HePNP immobilized on Ep-AG.

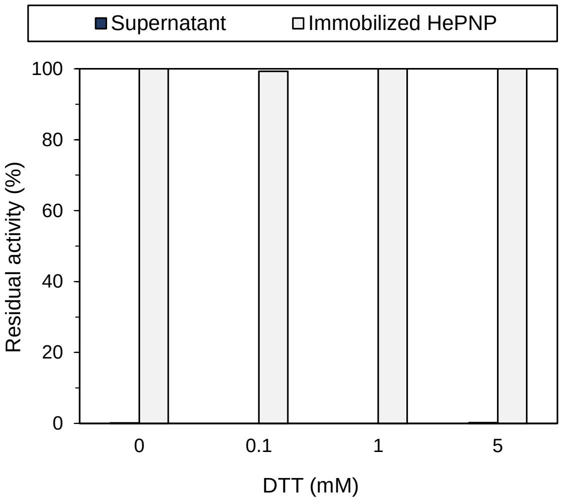

Figure S9. Stability of the binding chemistry between the (6x)Cystagged HePNP and the SH-AG. The activity of the immobilized enzyme and the activity of the supernatant after incubation with DTT for 24 h were measured. The activities are related to the control sample (0 mM DTT).

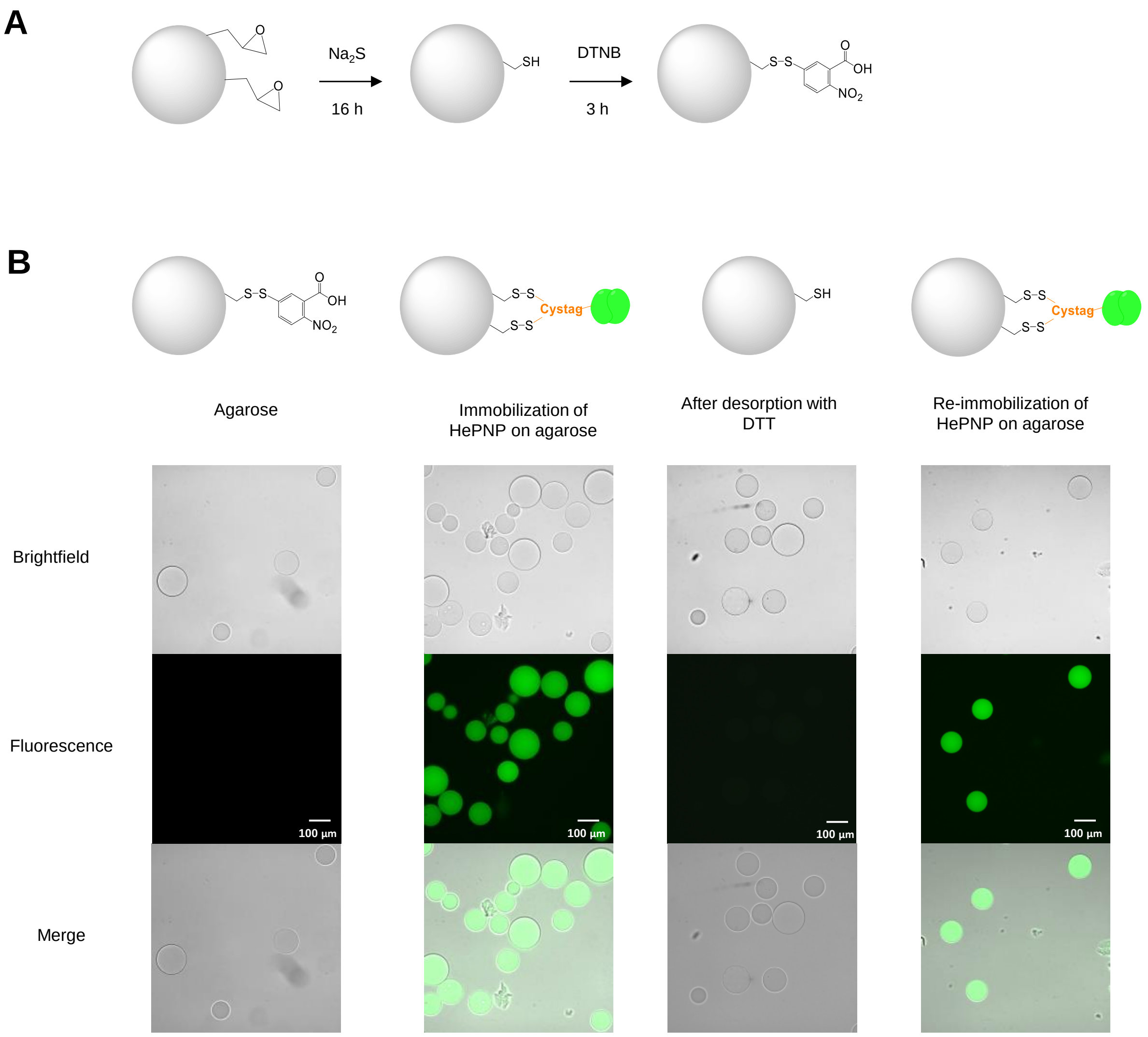

Figure S10. A) Scheme of the functionalization procedure of SH-AG (thiol-agarose) starting from Ep-AG (epoxy-agarose). **B)** Fluorescence microscopy imaging of FITC-labeled HePNP (green) immobilized on SH-AG trough

disulfide bonds between the (6x)Cys-tag (orange) of the HePNP and the thiols on the agarose beads. At the top the schemes show the binding chemistry between the HePNP (green) and the functionalized agarose beads (SH-AG, grey). At the bottom, the images obtained with the microscope. The enzyme was labeled with the fluorescein thiocyanate (FITC) as previously described.[1] The images were taken using a Nikon Ti2 Eclipse confocal microscope with a X-light V2 spinning disk. Objective 20x was used. λ_{ex}: 470 nm; emission filter 515 nm.

A

Your protein aproximate dimensions in Angstroms are: 93.479A, 51.87A, 92.131A. Which makes an aproximate volume of: 446720.66 A³\n
As for its surface, it measures 134728 A²\n

From which each amino acid contributes to:

ALA	7.54%
ARG	18.88%
ASN	0.83%
ASP	13.88%
CYS	0.00%
GLU	9.53%
GLN	0.00%
GLY	3.46%
HIS	1.92%
ILE	3.47%
LEU	6.80%
LYS	3.07%
MET	2.71%
PHE	6.09%
PRO	2.74%
SER	3.18%
THR	4.36%
TRP	0.00%
TYR	4.16%
VAL	7.35%

B

Your protein aproximate dimensions in Angstroms are: 136.033A, 93.744995A, 145.682A. Which makes an aproximate volume of: 1857797.1 A³\n
As for its surface, it measures 172033 A²\n

From which each amino acid contributes to:

ALA	6.68%
ARG	13.40%
ASN	0.98%
ASP	10.87%
CYS	3.10%
GLU	8.09%
GLN	1.49%
GLY	5.19%
HIS	6.91%
ILE	2.95%
LEU	6.22%
LYS	2.54%
MET	4.72%
PHE	4.51%
PRO	2.95%
SER	6.48%
THR	3.98%
TRP	0.15%
TYR	2.37%
VAL	6.43%

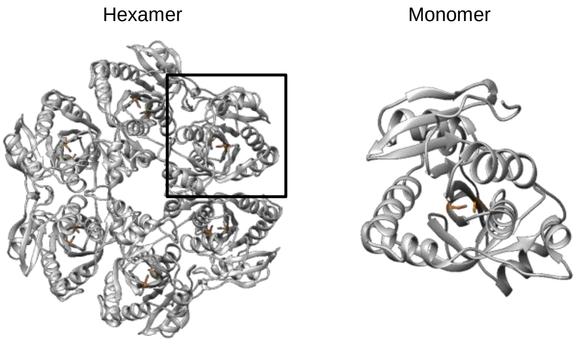

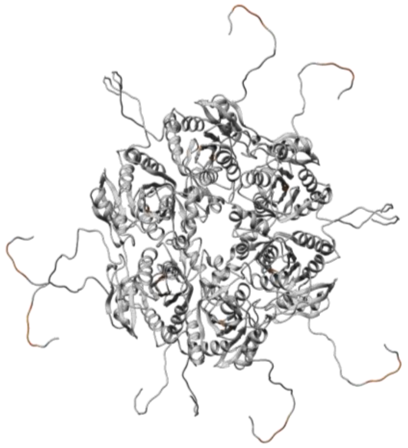

Figure S11. Analysis of the HePNP structure by CapiPy.[2] At the top, the protein and surface analysis performed by CapiPy. At the bottom, the model structures of HePNP by Pymol. **A)** Analysis of the native structure of HePNP. Twelve cysteine residues (two per monomer) are represented in orange. **B)** Analysis of structure of HePNP containing the (6x)Cys-tag at the N-terminal and the (6x)His-tag at the C-terminal. The six (6x)Cys-tag are represented in orange.

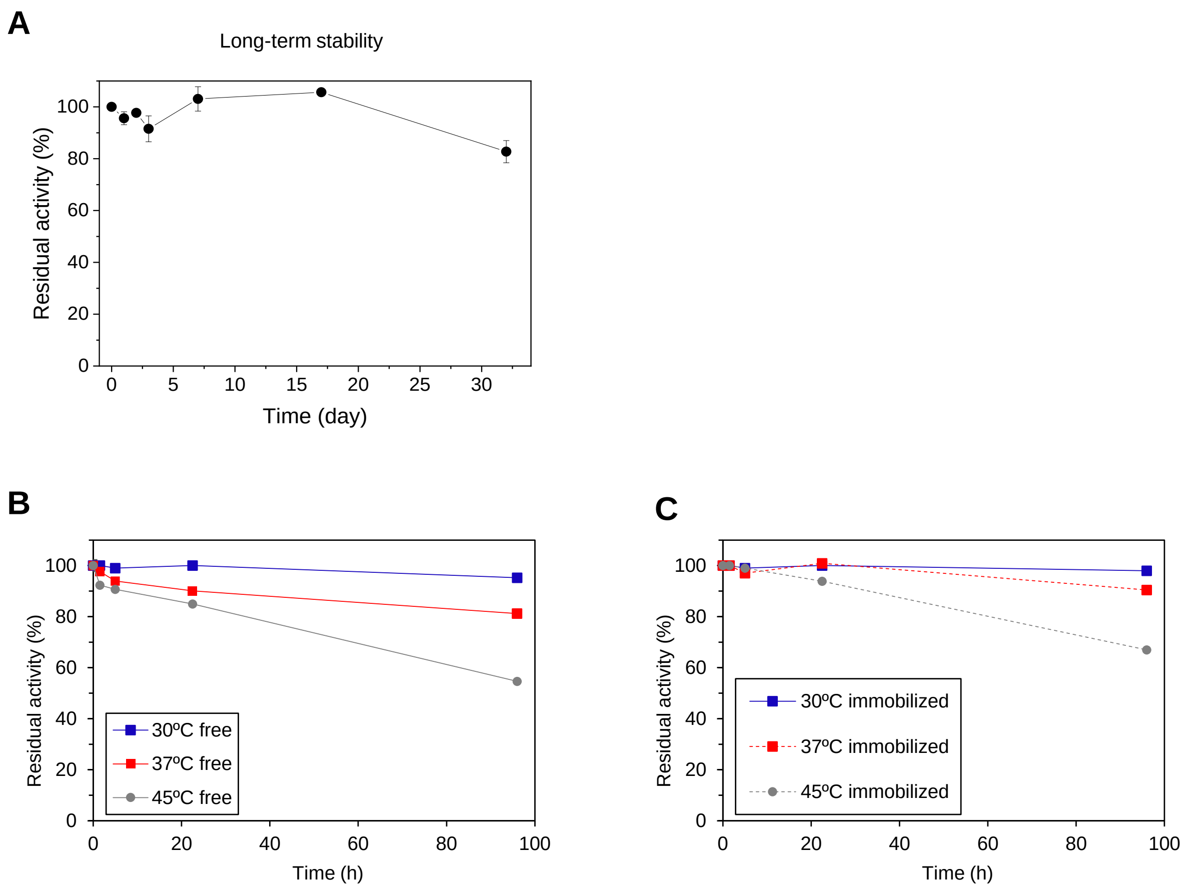

Figure S12. A) Long-term stability of HePNP. **B)** Stability of free (0.5 mg/mL) at different temperatures. **C)** Stability of immobilized (1 mg/g) HePNP on Ep-AG at different temperatures. The initial specific activity of the free enzyme corresponds to 20 U/mg while for the immobilized enzyme is 7 U/$g_{support}$. The preparations were incubated in 100 mM phosphate buffer pH 7.5

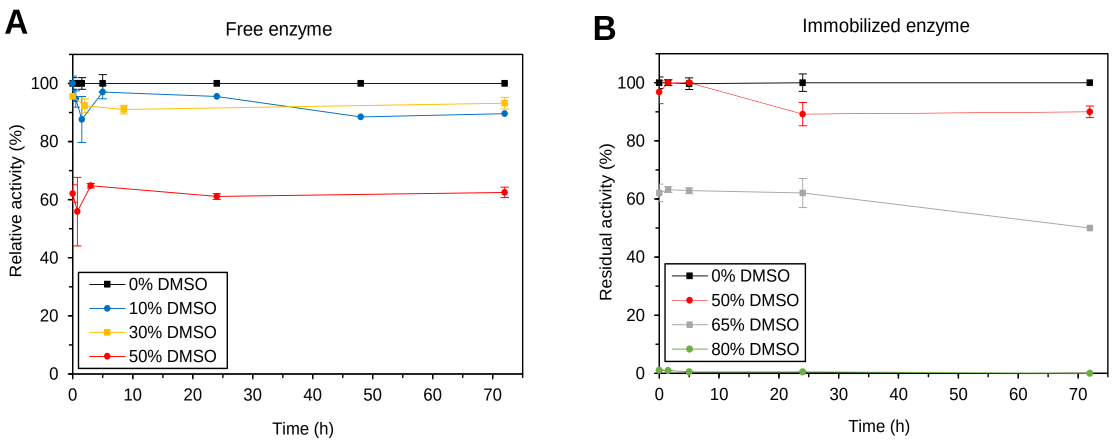

Figure S13. Stability towards DMSO of **A)** free HePNP (0.5 mg/mL) and **B)** immobilized HePNP on Ep-AG (100 mg in 1mL). The initial specific activity of the free enzyme corresponds to 20 U/mg while for the immobilized enzyme is 7 U/$g_{support}$**.** The preparations were incubated in 100 mM phosphate buffer pH 7.5 at room temperature.

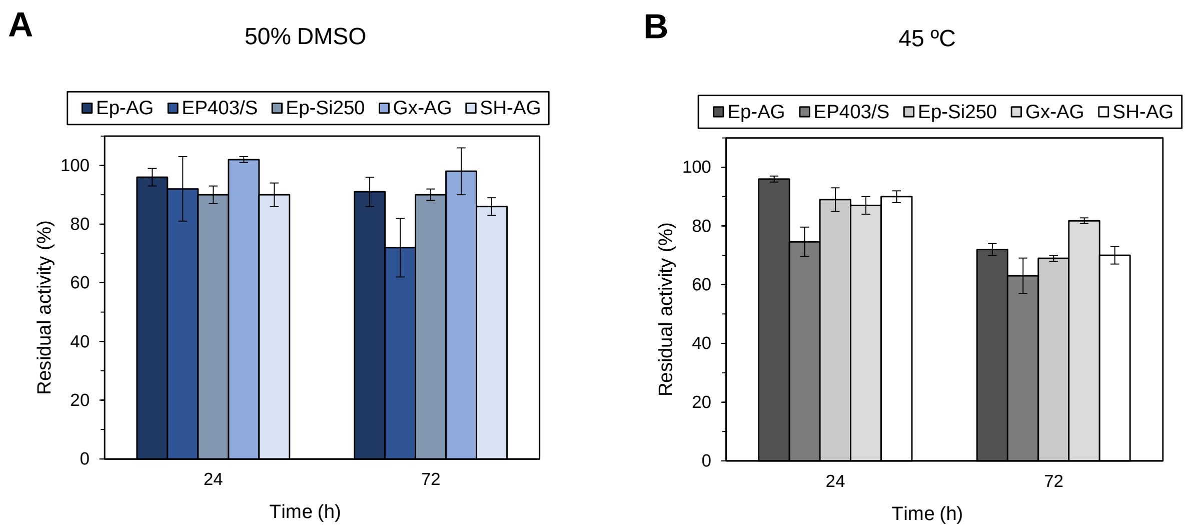

Figure S14. Stability of the immobilized HePNP on different supports at **A)** high concentration of solvent and **B)** high temperature. The initial activity of each support is reported in the Table 2. For all the experiments, 50 mg of the immobilized enzyme on each support were incubated in 0.5 mL of 100 mM phosphate buffer pH 7.5 (containing the corresponding DMSO).

Table S2. Effect of phosphate buffer concentration on the glycosylation efficiency. The biotransformations were performed with 50 mg of 1 mg/g immobilized HePNP in 1 mL of 5 mM of 6-O-methylguanine and 25 mM of inosine in phosphate buffer pH 7.5. The reactions were incubated at 37ºC for 24 h.

Phosphate	Phosphorolysis (%)	Transglycosilation (%)
1 mM	13	28
5 mM	19	52
10 mM	25	75
20 mM	33	85
50 mM	35	84
100 mM	41	80
500 mM	58	67

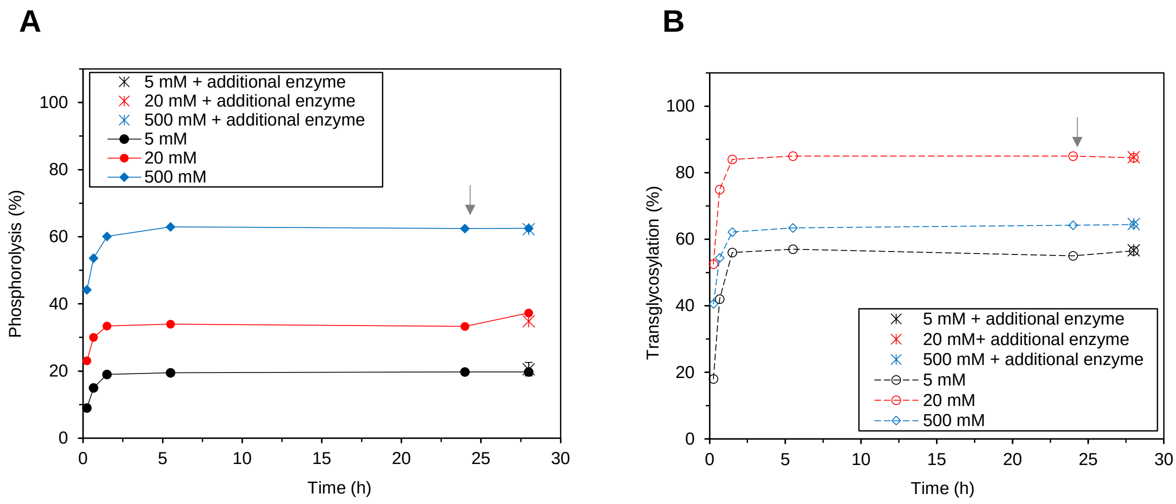

Figure S15. Equilibrium of the batch reactions at different concentrations of phosphate buffer (5, 20 and 500 mM). **A)** phosphorolysis conversion (%) and **B)** transglycosylation conversion (%) were monitored. The biotransformations were performed with 2 units HePNP in 1 mL of 5 mM of 6-O-methylguanine and 25 mM of inosine in phosphate buffer pH 7.5. The reactions were incubated at 37ºC for 28 h. After 24 h, one of the replicates was supplemented with additional enzyme (grey arrow).

Table S3. Effect of sugar donor (inosine) concentration on the glycosylation efficiency. The biotransformations were performed with 50 mg of 5 mg/g immobilized HePNP in 1 mL of 5 mM of 6-O-methylguanine in 20 mM phosphate buffer pH 7.5. The reactions were incubated at 37ºC for 24 h.

Sugar donor	Base	Phosphorolysis (%)	Hypoxantine (mM)	Transglycosylation (%)	Product (mM)
10 mM (2 eq.)	5 mM	53	5.3	70	3.5
20 mM (4 eq.)		48	9.4	78	3.9
30 mM (6 eq.)		36	11	82	4.1
40 mM (8 eq.)		32	12	85	4.3
50 mM (10 eq.)		26	13	90	4.5
100 mM (20 eq.)		21	22	99	5

Table S4. Optimization of the retention time (R.T.), temperature and sugar donor (inosine) concentration of the continuous flow reactions. The PBR contained 4 mg/g immobilized HePNP in 1.7 mL of column volume. The substrate mixture was 5 mM of 6-O-methylguanine and 25 mM inosine in 20 mM phosphate buffer pH 7.5, and was pumped at 0.85 mL/min, unless otherwise specified. The reactor was incubated at 37ºC unless otherwise specified.

R.T. (min)	Flow-rate (mL/min)	m.c. (%)
1	1.7	54.9 ± 0.4
2	0.85	77.9 ± 0.9
5	0.34	77.9 ± 0.2
10	0.17	75.2 ± 2.1
30	0.057	78.2 ± 1.5

Inosine (mM)	m.c. (%)
10 (2 eq.)	77.9 ± 0.9
25 (5 eq.)	89.5 ± 0.3
50 (10 eq.)	93.6 ± 0.2
90 (18 eq.)	>99

Temperature (ºC)	m.c. (%)
30	76.8 ± 1.1
37	87 ± 0.3
45	88.1 ± 0.4

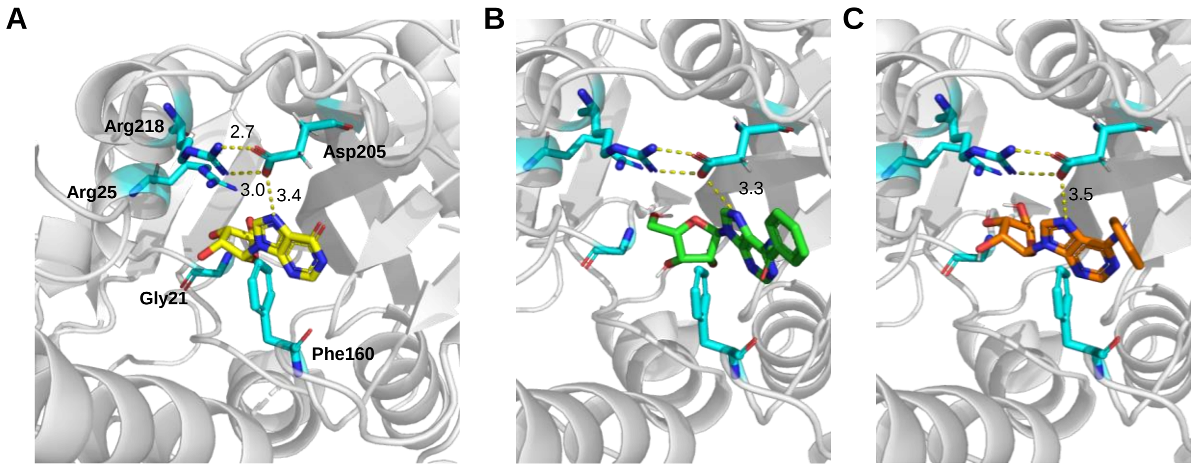

Figure S16. Docking analysis of A) inosine (yellow), B) N6-phenyl-adenosine (green), and C) N6-benzoyl-adenosine (orange). The residues implicated in the catalysis are highlighted in cyan. The distances between the N7 of the nucleobase and the Asp205 are stressed with yellow dashed lines. The distances between the Asp205 and the Arg218 are also stressed with yellow dashed lines.

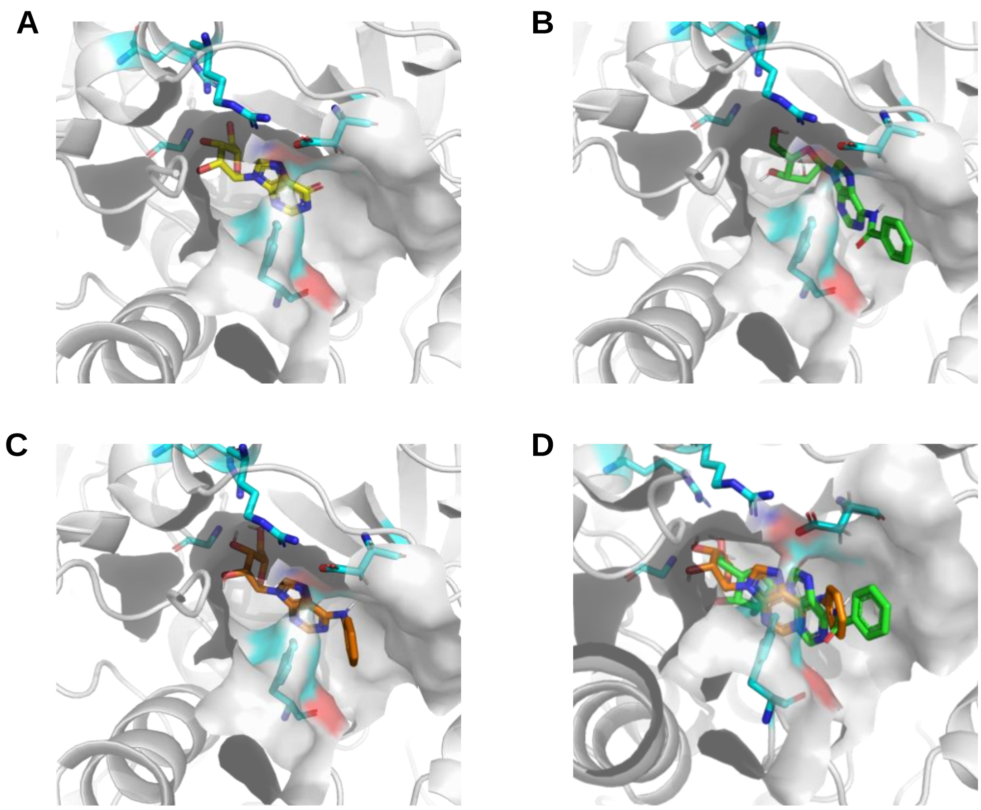

Figure S17. Docking analysis of A) inosine (yellow), B) N6-benzoyl-adensoine (green), C) N6-phenyl-adenosine (orange), and D) superposition of N6-phenyl-adenosine (orange) and N6-benzoyl-adensoine (green). The residues implicated in the catalysis are highlighted in cyan.

Table S5. Optimization of the sugar donor (inosine) concentration, temperature and phosphate concentration for the reactions using N6-phenyl-adenosine and N6-benzoyl-adenosine as nucleobase. The conversions of transglycosylation (%) are shown. The biotransformations were carried out in 2 mL tubes containing free enzyme. The experiments were performed with 5 mM of nucleobase and 20 eq. of sugar donor at 37 ºC and 20 mM phosphate buffer at pH 7.5 unless otherwise specified.

**Inosine (mM)**	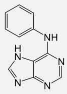	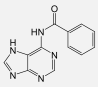
10 (2 eq.)	35.2	32.2
25 (5 eq.)	38.1	31.6
50 (10 eq.)	39.9	30.9
100 (20 eq.)	40.0	31.0

**Temperature (ºC)**	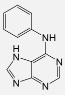	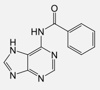
30	21.9	14.4
37	40.0	31.0
45	69.0	59.7

**Phosphate (mM)**	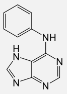	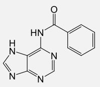
20	40.0	31.0
50	37.3	28.3
100	34.8	28.6

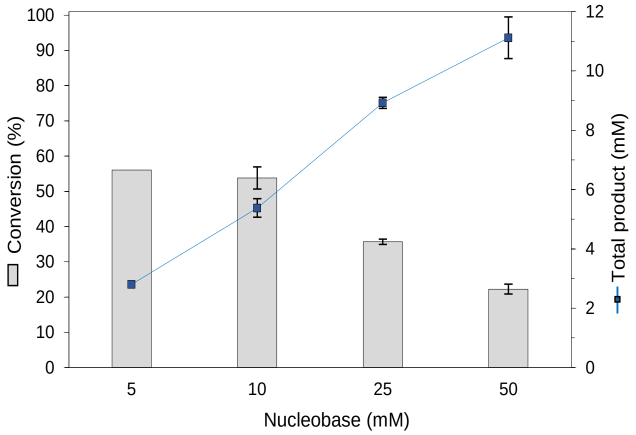

Figure S18. Optimization of the nucleobase (6-O-methylguanine) concentration in flow. 2 equivalents of inosine (10 mM, 20 mM, 50 mM, 100 mM) and 4 equivalents of phosphate buffer at pH 7.5 were used to feed the PBR. 5% and 10% of DMSO were added for the reactions at 25 mM and 50 mM, respectively. R.T. 2 min. Temperature: 37ºC. Flow-rate: 0.85 mL/min.

Table S6. Raw data of E-factor calculations. Mass of all reactants was calculated for each reaction cycle over 100 cycles under the conditions described in Table 4.

			**Substrates**		**Products**			
		**reagents**	Inosine	6-O-methylguanine	Hypoxantine	Nelarabine analogue	Phosphate	Water
		**Mw (g/mol)**	268.22	165.15	136.11	297.2	174.18	18
		**mM**	180	10			50	1
		**Volume (mL)**	1.7	1.7	1.7	1.7	1.7	1.7
**Cycles**	**Phosphorolysis**	**Transglycosylation**	**Reactants (µmol)**					
1	0.30	0.99	214.20	0.15	91.80	16.85	85	1.7
20	0.29	1.00	4116.31	0.07	1697.69	321.71	1615	32.3
40	0.29	1.00	4357.44	0.03	1762.56	339.32	1700	34
60	0.29	1.00	4332.96	0.05	1787.04	338.98	1700	34
80	0.29	1.00	4357.44	0.03	1762.56	339.32	1700	34
100	0.29	1.00	4351.32	0.09	1768.68	338.30	1700	34
		**Total (µmol)**	21729.67	0.43	8870.33	1694.48	1700	170
		Total mass	5.83	0.00	1.21	0.50	0.30	0.00
		Total E-factor	**14.6**					
		Total E-factor recycling inosine and buffer	**4.2**					

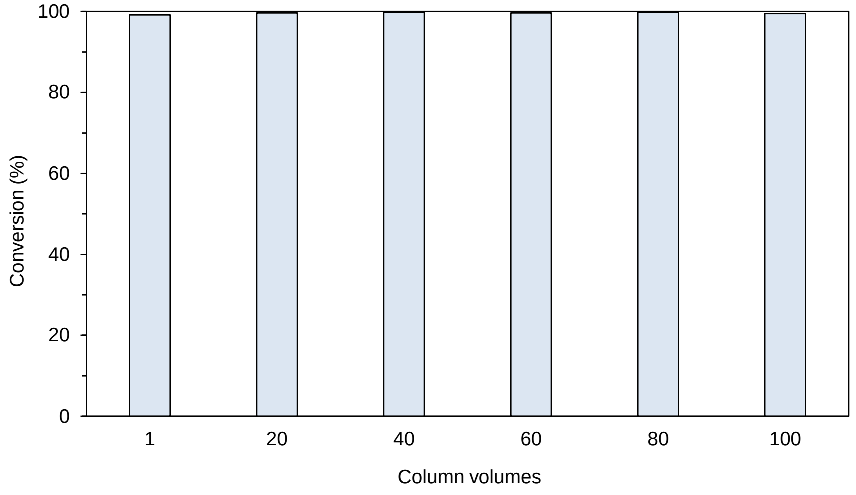

Figure S19. Operational stability of the immobilized HePNP on Ep-AG under flow conditions at 37ºC with R.T. 2 min and a flow-rate of 0.85 mL/min.

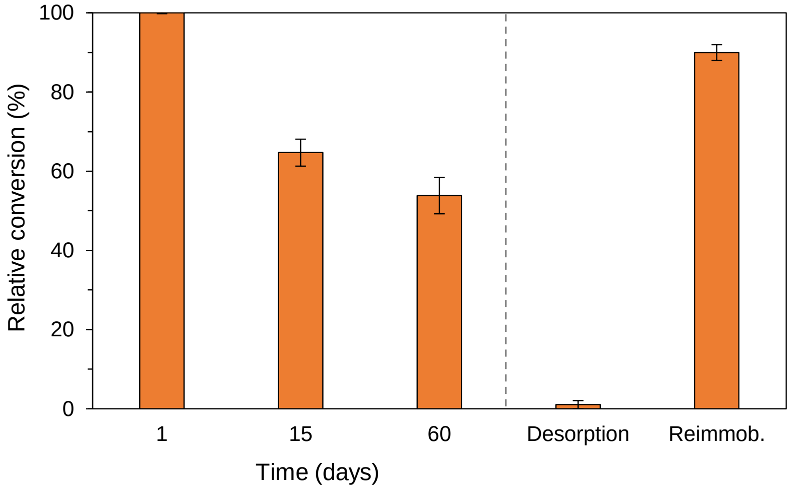

Figure S20. Stability after cycles of operation and storage of the HePNP immobilized on SH-AG and after the reuse of the support material. The PBR was used semi-continuously in flow, and in between the reactions it was stored at 4ºC. The desorption of the enzyme was performed with 50 mM DTT. The re-immobilization of the enzyme was carried out by offering the same amount of enzyme as immobilized before 4 mg/g (90% immobilization yield and 19% of recovered activity). The total relative conversion of the day one corresponds to 94.9% of m.c. The conversions were calculated by using a substrate solution of 10 mM inosine and 5 mM 6-O-methylguanine in 20 mM phosphate buffer at pH 7.5. R.T. 2 min. Temperature: 37ºC. Flow-rate: 0.85 mL/min.

Supporting references

[1] A. I. Benítez-Mateos, in *Methods Mol. Biol. (Immobilization of Enzymes and Cells)*, Humana Press Inc., **2020**, 20: 309-318.

[2] D. Roura Padrosa, V. Marchini, F. Paradisi, *Bioinformatics* **2021**, 37: 2761-2762.
